# Death of Dr. C. K. Gregg

**Published:** 1888-10

**Authors:** 


DEATH OF DR. C. K. GREGG.—This estimable gentleman and skilled physician met his death under distressing circumstrnces; he fell a victim to his ardor in scientific investigation. He had removed from Saltillo, Mexico, where he had resided for several years, to McKinney, Texas, only a few months ago. While engaged in testing and recording the effects of cocaine on his own person, it is supposed the drug produced mania, or temporary insanity, during which he put a pistol to his temple and fired, taking his own life. Nothing could have been further from Dr. Gregg's mind than suicide, during his rational existence. This case should be a warning to others. Dr. G. was a son of the venerable and beloved Bishop of Texas, and was literally an "ornament to his profession." He was quite a young man, but had a wide reputation as a physician.

